# Protective effect of early low-dose hydrocortisone on ventilator-associated pneumonia in the cancer patients: a propensity score analysis

**DOI:** 10.1186/s13613-017-0329-7

**Published:** 2017-10-23

**Authors:** David Lagier, Laura Platon, Jérome Lambert, Laurent Chow-Chine, Antoine Sannini, Magali Bisbal, Jean-Paul Brun, Karim Asehnoune, Marc Leone, Marion Faucher, Djamel Mokart

**Affiliations:** 1 Intensive Care Unit, Paoli-Calmettes Institute, 232 Boulevard de Sainte-Marguerite, 13009 Marseille, France; 2 Biostatistics Department, Saint Louis Teaching Hospital, AP-HP, 1, Avenue Claude Vellefaux, 75010 Paris, France; 3 Department of Anesthesiology and Critical Care Medicine, Hotel Dieu, University Hospital of Nantes, 1 Place Alexis Ricordeau, 44903 Nantes, France; 4 Department of Anesthesiology and Critical Care Medicine, Hopital Nord, University Hospital of Marseille, Chemin des Bourrely, 13015 Marseille, France

**Keywords:** Ventilator-associated pneumonia, Neoplasms, Immunomodulation, Hydrocortisone, Propensity score

## Abstract

**Background:**

Ventilator-associated pneumonia (VAP) is a care-related event that could be promoted by immune suppression caused by critical diseases, malignancies and cancer treatments. Low dose of hydrocortisone was proposed for modulation of immune response in the critically ill population.

**Methods:**

In this monocentric observational study, all cancer patients mechanically ventilated for more than 48 h were included. Effect of low-dose hydrocortisone administered during the first 48 h of mechanical ventilation was evaluated applying inverse probability weighting analysis after propensity score assessment. VAP impact on 1-year mortality, ICU length of stay and mechanical ventilation duration was secondarily determined.

**Results:**

Within this cohort, 190 cancer patients were followed. VAP was confirmed in 22.1% of cases in the early hydrocortisone group and confirmed in 42.6% of cases in the no or late hydrocortisone group. Early hydrocortisone exhibited a protective effect on the risk of VAP (OR 0.23; 95% CI 0.12–0.44; *P* < 0.0001). VAP was associated with 1-year mortality (HR 1.60; 95% CI 1.10–2.34; *P* = 0.017) and increased ICU length of stay (mean extra length of stay: 4.2 days; 95% CI 0.6–7.8).

**Conclusions:**

Immune modulation with low-dose hydrocortisone administered in the first days of mechanical ventilation could protect from VAP occurrence in cancer patients.

## Background

Recently introduced aggressive treatments have significantly decreased the overall mortality rate in cancer patients [1]. These new approaches come at the price of a steep rise in infections and treatment-related toxicities [2]. Immune suppression with or without neutropenia is a major concern in this setting. On the other side, critical conditions found during sepsis or acute respiratory failure induce a complex immune response making severely ill patients prone to secondary ICU-acquired infections, such as ventilator-associated pneumonia (VAP) [3]. During sepsis, hydrocortisone improves the phagocytic abilities of neutrophils, decreases the blood concentration of anti-inflammatory cytokines (interleukin-10) and increases the blood concentrations of the host defence against infection (interferon γ and interleukin-12) [4, 5]. By balancing the inflammatory response, hydrocortisone might also decrease the growth and virulence of bacteria [6, 7]. In septic shock, low-dose hydrocortisone improves shock reversal irrespective to adrenal response to corticotropin [8]. Moreover, it has been shown that low-dose hydrocortisone can reduce the incidence of hospital-acquired pneumonia in intubated patients with multiple trauma [9]. Survival of cancer patients with acute respiratory failure has improved over time to about 60% [10]. Nevertheless, invasive mechanical ventilation remains associated with a 28-day mortality rate of about 50% [11]. In non-selected populations, VAP is a common hospital-acquired pneumonia and occurs in up to 30% of patients receiving mechanical ventilation for more than 48 h. The main objective of our study was to evaluate the preventive role of early treatment with low-dose hydrocortisone regarding incidence of VAP in cancer patients. The prognostic impact of VAP on 1-year mortality, mechanical ventilation duration and ICU length of stay was secondarily assessed.

## Methods

### Study population

In this monocentric observational study, all consecutive cancer patients requiring invasive mechanical ventilation for more than 48 h that have been admitted to our ICU between January 1, 2009, and December 31, 2013, were prospectively followed. We excluded from the study patients that needed two or more invasive mechanical ventilation periods during their ICU stay. The Paoli-Calmettes Institute Institutional Review Board approved this observational study (No. IPC-2017-077). No consent was needed in this observational study.

### Diagnosis of VAP

All ventilated patients were daily screened for new respiratory or septic events. VAP was suspected if a recent and persistent infiltrate on chest radiograph was associated with at least two of the following criteria: hyperthermia (> 38 °C) or hypothermia (< 36 °C), purulent tracheal secretions and worsening of gas exchange. Because of its high variability and poor specificity in the onco-haematological context, leucocyte count was not taken into account. Quantitative microbiological culture of 10^6^ colony-forming unit (CFU)/mL of a typical pathogen from endotracheal aspirate or 10^4^ CFU/mL from bronchoalveolar lavage fluid confirmed VAP [12, 13]. Early VAP was defined as a VAP diagnosed before the 5th day of invasive mechanical ventilation. An adjudication committee (two senior ICU physicians) systematically reviewed VAP diagnosis to determine whether it meets protocol-specified criteria. It was blinded to hydrocortisone status.

### VAP bundles

In our ICU, VAP prevention strategy included 30° semi-sitting position, endotracheal cuff pressure control, chlorhexidine 0.2% daily oral care and a sedation protocol based on the Richmond Agitation Sedation Scale with daily sedation discontinuation. No selective digestive or oropharyngeal decontamination was used. Enteral nutrition was gradually implemented as early as possible. Parenteral nutrition was used if contraindication or poor tolerance to enteral route was present. Anti-acid treatment was pursued during mechanical ventilation periods irrespective of the hydrocortisone status.

### Low-dose hydrocortisone treatment

In this study, low-dose hydrocortisone was usually prescribed in case of refractory septic shock with persistent arterial hypotension despite high-dose vasopressor therapy (≥ 0.8 μg kg^−1^ min^−1^ of norepinephrine) or as an alternative therapy in case of sepsis with previous curative corticosteroid therapy. In case of sepsis and ongoing curative corticosteroid therapy, the treatment was switched for hydrocortisone. Fifty milligrams was administered intravenously every 6 h according to our local protocol.

### Data collection

All data were extracted and analysed by senior physicians using our ICU management software (MetaVision ICU, iMDsoft Inc.^®^, Dedham, MA, USA). As previously described [10], baseline data were recorded upon ICU admission: gender, age, cancer type, cancer stage classified in four categories (newly diagnosed, complete remission, partial remission and evolutive disease), main ICU admission purpose (septic shock, acute respiratory failure, coma and others), presence of neutropenia, history of haematopoietic stem cell transplantation (HSCT) and recent exposure to antibiotics or curative corticosteroids (during the 10 days before admission). SOFA score [14] was also reported at the time of endotracheal intubation. Several approaches implemented during the first 48 h after endotracheal intubation, including vasopressors, renal replacement therapy, substitutive steroids therapy for refractory shock, granulocyte colony-stimulating factors (G-CSF), enteral nutrition and antibiotherapy (adapted or empirical), were recorded. VAP microbiological evidences were also documented. ICU mortality was evaluated. ICU survivors were prospectively followed after ICU discharge until the end of the study and 1-year survival was determined.

### Statistical analysis

Data are presented as median (interquartile range) for quantitative variables and count (percentages) for qualitative variables. Binary outcome (i.e. the occurrence of VAP) was analysed using a Chi-square test or the nonparametric Wilcoxon rank-sum test as appropriate. The multivariate analyses were performed using a logistic model. The primary outcome of the study was to evaluate the prevention of VAP using early low dose of hydrocortisone. VAP incidence was reported to the incidence per 1000 ventilator days. Effect of early low-dose hydrocortisone on incidence of VAP was studied using propensity score analysis to take into account the non-randomized design of this study. Early hydrocortisone group was defined by hydrocortisone treatment initiated during the first 48 h of invasive mechanical ventilation. Patients treated by hydrocortisone for more than 48 h before tracheal intubation were excluded from this analysis. Propensity score, which is the probability that a patient will receive low-dose hydrocortisone, was assessed using a logistic regression model with baseline covariates as explanatory variables and treatment with low-dose hydrocortisone as the outcome. An inverse probability weighting (IPW) analysis was then performed to assess the average treatment effect of low-dose hydrocortisone assessed by comparison of two pseudo-population, one where nobody would have received low-dose hydrocortisone and one where everybody would have received it. Cumulative incidence of VAP in ICU was estimated taking into accounts competing risk of discharge of ICU (either death or discharge alive).

Association between baseline variables, describing patient’s condition at ICU admission or at intubation, and overall mortality was assessed by univariable analysis using Cox proportional hazard models. Multivariable analysis including variables significantly associated with death was performed using a Cox proportional hazard model with VAP as a time-dependent variable. Variable selection was based on Akaike information criteria (AIC). Since VAP is a time-dependent event, it cannot be treated as a baseline covariate. Hence, a Mantel–Byar analysis was performed to assess and graphically display the effect of VAP on 1-year mortality. To estimate extra length of stay (in ICU-discharged patients) and extra duration of intubation (in extubated patients) due to VAP, we used a multistate model that takes into account time to VAP.

## Results

Between January 1, 2009, and December 31, 2013, 208 patients were included in the study. Among them, 18 have been excluded for multiple periods of invasive mechanical ventilation. Among the 190 patients included in the final analysis, 55 (28.9%) develop a confirmed VAP. Early VAP onset was found in 12 patients (21.8% of the total VAP). Microbiological data are outlined in Table 1. Substitutive corticotherapy with low-dose hydrocortisone was prescribed in 122 (64.2%) cases and was predominantly used in patients without VAP (*P* = 0.003; Table 2). The median mechanical ventilation duration was 11 (6–18) days. ICU and 1-year mortality rate were 56 and 77%, respectively (Table 3).

**Table 1:** Microbiological documentation depending on the timing of VAP

	Early VAP (*n* = 12)	Late VAP (*n* = 43)
*P. aeruginosa*	4	10
*E. coli*	2	6
*K. pneumoniae*	1	6
*E. cloacae*	1	3
*Enterococcus sp*	2	8
*Staphylococcus sp*	1	2
*Stenotrophomonas sp*	0	5
Other Gram-negative bacteria	1	3

**Table 2:** Patient’s characteristics

Variables	Patients without VAP (*n* = 135)	Patients with VAP (*n* = 55)	*P*
Male gender, *n* (%)	87 (64.4)	41 (74.5)	0.23
Age (year), median (IQR)	59.2 (52.2–65.8)	60.4 (50.2–67.1)	0.99
Cancer type			0.39
Haematological malignancy, *n* (%)	95 (70.4)	35 (63.6)	
Solid tumour, *n* (%)	40 (29.6)	20 (36.4)	
Cancer stage			0.91
Diagnosis, *n* (%)	36 (26.7)	15 (27.3)	
Complete remission, *n* (%)	29 (21.5)	13 (23.6)	
Partial remission, *n* (%)	31 (23)	14 (25.5)	
Evolutive, *n* (%)	39 (28.9)	13 (23.6)	
HSCT, *n* (%)	47 (24.7)	14 (25.4)	0.43
Admission purpose			0.32
Septic shock, *n* (%)	59 (43.7)	21 (38.2)	
Acute respiratory failure, *n* (%)	51 (37.8)	27 (49.1)	
Coma, *n* (%)	14 (10.4)	2 (3.6)	
Others, *n* (%)	11 (8.1)	5 (9.1)	
Clinical sepsis upon admission			0.042
Respiratory, *n* (%)	79 (58.5)	31 (56.4)	
Non-respiratory, *n* (%)	31 (23)	6 (10.9)	
None, *n* (%)	25 (18.5)	18 (32.7)	
Characteristics upon admission			
Neutropenia, *n* (%)	54 (40)	18 (32.7)	0.41
Antibiotherapy, *n* (%)	104 (77)	43 (78.2)	1
Corticosteroids (curative), *n* (%)	32 (23.7)	19 (34.5)	0.15
SOFA score (day of intubation), median (IQR)	11 (8–14)	11 (8–13)	0.28
Characteristics at the first 48 h of MV			
Vasopressors, *n* (%)	99 (73.3)	44 (80)	0.36
Renal replacement therapy, *n* (%)	28 (20.7)	9 (16.4)	0.55
Substitutive hydrocortisone, *n* (%)	96 (71.1)	26 (47.3)	0.003
G-CSF, *n* (%)	20 (14.8)	9 (16.4)	0.82
Enteral nutrition, *n* (%)	40 (29.6)	21 (38.2)	0.3
Antibiotherapy			0.09
Adapted, *n* (%)	41 (30.4)	11 (20)	
Empirical, *n* (%)	89 (65.9)	38 (69)	
None, *n* (%)	5 (3.7)	6 (10.7)	

*HSCT* haematopoietic stem cell transplantation, *G-CSF* granulocyte colony-stimulating factors, *MV* mechanical ventilation, *IQR* interquartile range

**Table 3:** Predictors of 1-year mortality: univariate and multivariate analysis

Variables	Univariate			Multivariate		
	HR	95% CI	*P*	HR	95% CI	*P*
Male gender	0.89	0.64–1.24	0.49			
Age	0.99	0.98–1	0.13			
Cancer type						
Haematological malignancy	1	(Reference)	0.91			
Solid tumour	0.91	0.65–1.28				
Cancer stage						
Complete remission	1	(Reference)	0.02	1	(Reference)	0.03
Diagnosis	1.37	0.87–2.16		1.44	0.88–2.35	
Partial remission	0.82	0.50–1.33		0.98	0.57–1.68	
Evolutive	1.54	0.99–2.41		1.77	1.10–2.87	
HSCT	1.28	0.86–1.92	0.23			
Admission purpose						
Others	1	(Reference)	0.055			
Septic shock	0.96	0.54–1.71				
Acute respiratory failure	0.62	0.34–1.11				
Coma	0.66	0.31–1.43				
Clinical sepsis upon admission						
None	1	(Reference)	0.02	1	(Reference)	0.04
Respiratory	1.67	1.09–2.56		1.65	1.03–2.65	
Non-respiratory	1.91	1.16–3.15		1.78	1.01–3.15	
Characteristics upon admission						
Neutropenia	1.41	1.02–1.94	0.04			
Antibiotherapy	1.18	0.80–1.72	0.4			
Corticosteroids (curative)	1.03	0.73–1.45	0.89			
SOFA score (day of intubation)	1.10	1.06–1.15	0.0001	1.11	1.05–1.17	0.0002
Characteristics at the first 48 h of MV						
Vasopressors	1.34	0.93–1.94	0.11			
Renal replacement therapy	1.45	1.00–2.12	0.06			
Substitutive hydrocortisone	1.28	0.92–1.77	0.28			
G-CSF	1.85	1.23–2.78	0.005	1.65	1.03–2.65	0.042
Enteral nutrition	1.17	0.68–2.00	0.58			
Antibiotherapy						
Adapted, *n* (%)	1	(Reference)	0.83			
Empirical, *n* (%)	0.85	0.6–1.22				
None, *n* (%)	0.96	0.5–1.86				
VAP	1.41	0.98–2.03	0.06	1.60	1.10–2.34	0.017

*HSCT* haematopoietic stem cell transplantation, *G-CSF* granulocyte colony-stimulating factors, *MV* mechanical ventilation, *VAP* ventilator-associated pneumonia

### Effect of early low-dose hydrocortisone

**Fig. 1:**
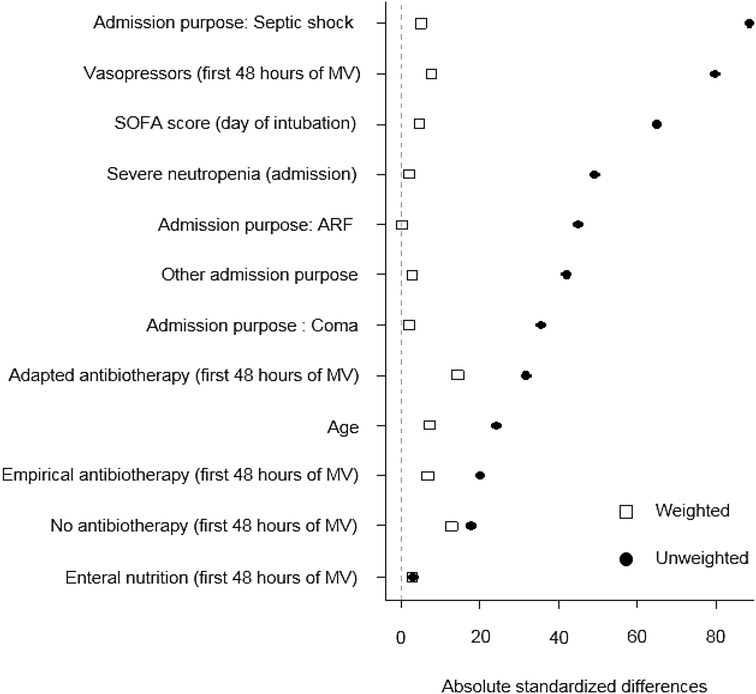
Covariate imbalance (assessed by standardized mean differences) between the two groups of patients receiving and not receiving early HC in the unweighted (original) and weighted populations

**Fig. 2:**
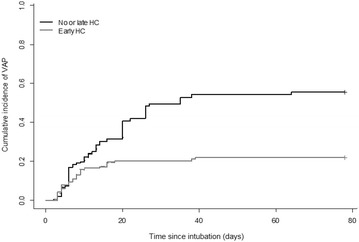
Cumulative incidence of ventilator-associated pneumonia in the inverse probability of treatment weighting analysis

Nine patients received hydrocortisone for more than 48 h before tracheal intubation and were excluded from this analysis. Global VAP incidence in the 181 patients included in the analysis; incidence was 25.5/1000 ventilator days. Stratified according to cortisone, incidence was 20.3/1000 ventilator days in the group receiving early low-dose hydrocortisone and 32.7/1000 ventilator days in the group receiving no or late low-dose hydrocortisone. A prior multivariable analysis has identified early hydrocortisone treatment as the only independent variable significantly associated with the VAP occurrence (OR 0.41; 95% CI 0.2–0.8; *P* < 0.01). The propensity score was constructed using the following relevant variables: age, neutropenia and admission purpose at admission, as well as SOFA score, vasopressors, antibiotherapy (adapted, empirical, none) and enteral nutrition at the time of intubation. Standardized differences in the unweighted population and in the weighted population are shown in Fig. 1. VAP was confirmed in 22.1% of cases in the early hydrocortisone group (25 out of 113 patients) and confirmed in 42.6% of cases in the no or late hydrocortisone group (29 out of 68 patients). Using IPW analysis, early hydrocortisone exhibited a protective effect on the risk of VAP (OR 0.23; 95% CI 0.12–0.44; *P* < 0.0001, Fig. 2).

### VAP prognostic impact

**Fig. 3:**
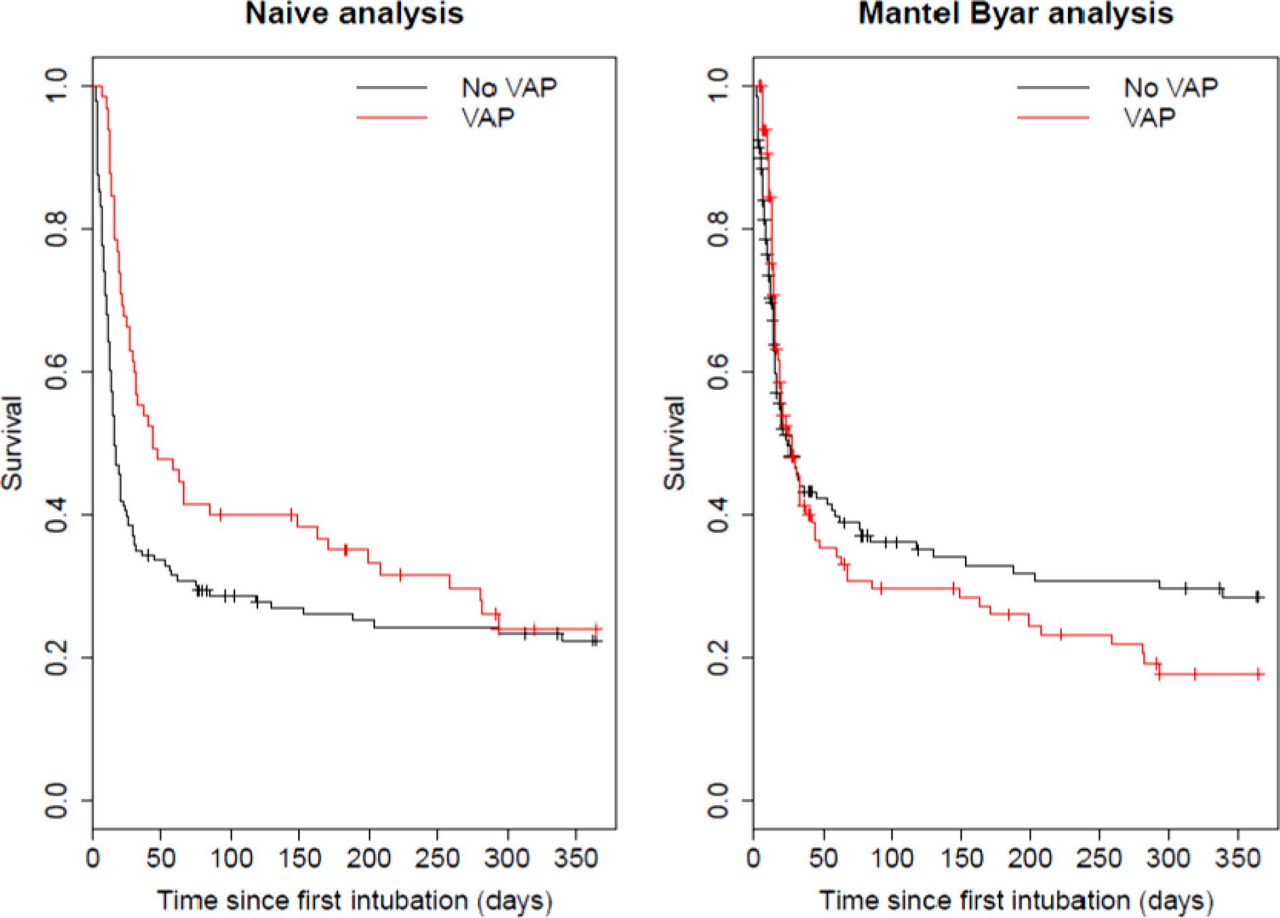
One-year survival according to ventilator-associated pneumonia status: naive analysis and time-dependent Mantel–Byar analysis

Considering VAP as a time-dependent covariate, univariate analysis (Table 2) revealed that VAP is not associated with 1-year mortality (HR 1.41; 95% CI 0.98–2.03; *P* = 0.06). After multivariate adjustment (Table 2), an independent and significant association is revealed between VAP and 1-year mortality (HR 1.60; 95% CI 1.10–2.34; *P* = 0.017, Fig. 3). Regarding initial vs late onset VAP, there indeed was a difference in prognosis, with late onset VAP being associated with a higher mortality [HR 1.74 (95% CI 1.17–2.58)], but early VAP being not significantly different from no VAP [HR 0.98 (0.39–2.46)]. VAP resulted in a significantly longer ICU stay for patients discharged [mean extra length of stay: 4.2 days (95% CI 0.6–7.8)] and a longer, although not significant, mechanical ventilation duration for patients extubated [mean extra duration: 1.7 days (95% CI − 1.5 to 5.0)].

## Discussion

We report herein on 190 onco-haematology patients admitted to ICU and treated with mechanical ventilation over 4 years. We showed the protective effect of low-dose hydrocortisone administered in the first days of mechanical ventilation regarding VAP occurrence. An association was found between VAP occurrence and 1-year mortality. The deleterious impact of VAP on ICU length of stay was also demonstrated. To our knowledge, this is the first study reporting prognosis data with regard to VAP in the specific cancer population.

VAP is a controversial topic [15–17]. In the global ICU population, VAP incidence and attributable mortality are uncertain [18, 19]. In a systematic review of published randomized trials [20], VAP-cumulated incidence varies from 9% to more than 40% depending on the study and the given population. This heterogeneity is mainly explained by the lack of consensual definition [17, 21] and by the variability in VAP bundles implementation rates [22, 23] in the different ICUs. In this study, we used an association of clinical and bacteriological criteria to diagnose VAP in reference to the 2005 ATS/IDSA guidelines [12]. This definition is slightly different from the definition of probable VAP according to the last CDC definition of ventilator-associated events [24], which emphasize on FiO2 and positive end-expiratory pressure adjustment in response to worsening oxygenation. However, the prognostic significance of that current CDC definition remains to be established [25]. The cumulative incidence of VAP in our cohort was 28.9%. Despite regular use of validated VAP bundles in our ICU, this remains relatively high. Cancer treatments and malignancy-related immunosuppression could explain the higher susceptibility to develop nosocomial infections in the onco-haematological population.

Theoretically, VAP is a delayed event that happens after 48 h of mechanical ventilation. The pathophysiology of nosocomial infections combines the bacterial colonization induced by the invasiveness of general ICU cares (endotracheal intubation, catheter, etc.) and a state of susceptibility to infection [26]. It is well recognized that initial aggression induces a delayed state of profound immunodeficiency few days after the initial insult [3, 27]. More specifically, a biphasic evolution of immunological competence has been well described in sepsis [28] and trauma [29]. After an initial pro-inflammatory phase, a post-aggressive phase is characterized by a compensatory systemic anti-inflammatory state and an apoptotic depletion of immune cells [30, 31]. This delayed immunological status confers wider susceptibility to ICU-acquired infection [28] and viral reactivation. In the haematology population, ICU-induced immunodeficiency has also been described in neutropenic patients [32]. Monocyte and alveolar macrophage deactivation have been described after septic ARDS [33, 34] and could thus facilitate the occurrence of ICU-acquired infections. In order to counteract this phenomenon, low dose of hydrocortisone has been suggested to prevent post-aggressive immunosuppression. Indeed, hydrocortisone improves immune capacities, decreases blood concentrations of anti-inflammatory cytokines (interleukin-10) and increases host defence cytokines (interferon γ and interleukin-12) [4, 5]. However, despite positive hemodynamic effect, beneficial effect of substitutive corticotherapy on septic shock survival remains controversial [8, 35, 36]. Last study showed that it failed to prevent the development of septic shock in the severe sepsis population [37]. The HIPOLYTE study [9] has compared low-dose hydrocortisone to placebo in the first 28 days after a severe trauma. It showed a reduction in the incidence of hospital-acquired pneumonia with 4 more ventilation-free days. Despite hydrocortisone treatment, no significant reduction in norepinephrine treatment duration was found in this study. To be effective, hydrocortisone should be started earlier as possible in order to decrease the initial pro-inflammatory response and counteract the anti-inflammatory compensation. In immunosuppressed cancer patients, we focused on potential beneficial effects of the early initiation of substitutive hydrocortisone for VAP prevention. A reverse propensity score analysis was used to control bias and population heterogeneity inherent to non-randomized observational studies. After weighing on the most pertinent covariates, we found that early hydrocortisone prescribed around the intubation time was protective against the subsequent occurrence of VAP.

VAP impact on mortality remains debated [38, 39]. The overuse of traditional crude statistical test in past studies had led to conflicting results and overestimation of attributable mortality of VAP [21]. In our work, VAP was not associated with mortality in the naive-exposed–unexposed analysis. That result is surely related to the high mortality rates in the first days of ICU admission in the critically ill cancer patients. Indeed, a majority of patients did not have time to develop VAP before dying (competing risk). On the contrary, by considering VAP as a time-dependent variable and estimating survival from the time of VAP diagnosis, we showed that VAP was significantly associated with 1-year mortality.

Our study has limitations. First, despite the use of propensity score analysis, this study is observational and residual confounding factors and biases may exist. For example, exposure to chemotherapy with or without curative corticotherapy before ICU stay has not been taking into account. However, neutropenic status was included in the baseline covariates for the propensity score construction. Second, this is a monocentric study, so it is possible that our local protocol including VAP prevention bundle, diagnosis strategies and therapeutic management could influence the occurrence and the prognostic impact of the disease. Third, adherence to VAP prevention bundle is not reported in each treatment group and could induce a bias. Finally, systemic antibiotic treatment could play a preventive role on VAP occurrence depending on its spectrum and its duration. These data are missing, but it is likely that the liberal use of broad spectrum antibiotics in the immunocompromised patients, irrespective of the hydrocortisone status, would diminish the confounding effect of these parameters.

## Conclusions

We found a positive effect of early low-dose hydrocortisone treatment in preventing VAP. Immunological aspects are crucial in the development of nosocomial infections, specifically in patients prone to immunological disorders. Critically ill cancer patients could benefit from the administration of low-dose hydrocortisone in the days surrounding mechanical ventilation initiation. This interesting result should be evaluated in a future large-scale randomized controlled trial.

## Abbreviations

CFU: colony-forming unit
G-CSF: granulocyte colony-stimulating factors
HSCT: haematopoietic stem cell transplantation
ICU: intensive care unit
IPW: inverse probability weighting
VAP: ventilator-associated pneumonia
